# Supplementary material for: Complete Chloroplast Genomes and Comparative Analyses of L. chinensis, L. anhuiensis, and L. aurea (Amaryllidaceae)
Source: Int J Mol Sci. 2020 Aug 10;21(16):5729. doi: 10.3390/ijms21165729 (PMC7461117; doi:10.3390/ijms21165729)
Supplement: Supplementary file 1 [file ijms-21-05729-s001.zip › ijms-876965-supplementary/Table S1 Splitting genes with introns and exons in the chloroplast genomes of seven Lycoris species.docx]

**Table S1.** Splitting genes with introns and exons in the chloroplast genomes of seven *Lycoris* species

| **Species** | **Gene** | **Location** | **Exon I (bp)** | **Intron I (bp)** | **Exon II (bp)** | **Intron II (bp)** | **Exon III (bp)** |
| --- | --- | --- | --- | --- | --- | --- | --- |
| *L. chinensis* | *trnK-UUU* | LSC | 46 | 2577 | 35 |  |  |
|  | *rps16* | LSC | 38 | 887 | 208 |  |  |
|  | *trnG-UCC* | LSC | 23 | 691 | 48 |  |  |
|  | *atpF* | LSC | 154 | 1069 | 257 |  |  |
|  | *rpoC1* | LSC | 432 | 750 | 1626 |  |  |
|  | *ycf3* | LSC | 126 | 725 | 231 | 755 | 153 |
|  | *trnL-UAA* | LSC | 30 | 517 | 50 |  |  |
|  | *trnV-UAC* | LSC | 39 | 584 | 37 |  |  |
|  | *clpP* | LSC | 71 | 788 | 291 | 658 | 250 |
|  | *petB* | LSC | 6 | 785 | 642 |  |  |
|  | *petD* | LSC | 8 | 737 | 514 |  |  |
|  | *rpl16* | LSC | 9 | 963 | 402 |  |  |
|  | *rpl2* | IR | 394 | 666 | 428 |  |  |
|  | *ndhB* | IR | 775 | 699 | 758 |  |  |
|  | *trnI-GAU* | IR | 42 | 935 | 35 |  |  |
|  | *trnI-GAU* | IR | 42 | 935 | 35 |  |  |
|  | *trnA-UGC* | IR | 38 | 815 | 35 |  |  |
|  | *trnA-UGC* | IR | 38 | 815 | 35 |  |  |
|  | *ndhA* | SSC | 559 | 1125 | 539 |  |  |
|  | *trnA-UGC* | IR | 38 | 815 | 35 |  |  |
|  | *trnA-UGC* | IR | 38 | 815 | 35 |  |  |
|  | *trnI-GAU* | IR | 42 | 935 | 35 |  |  |
|  | *trnI-GAU* | IR | 42 | 935 | 35 |  |  |
|  | *ndhB* | IR | 775 | 699 | 758 |  |  |
|  | *rpl2* | IR | 394 | 666 | 428 |  |  |
| *L. anhuiensis* | *trnK-UUU* | LSC | 46 | 2577 | 35 |  |  |
|  | *rps16* | LSC | 38 | 888 | 208 |  |  |
|  | *trnG-UCC* | LSC | 23 | 691 | 48 |  |  |
|  | *atpF* | LSC | 154 | 1069 | 257 |  |  |
|  | *rpoC1* | LSC | 432 | 750 | 1626 |  |  |
|  | *ycf3* | LSC | 126 | 725 | 231 | 755 | 153 |
|  | *trnL-UAA* | LSC | 30 | 517 | 50 |  |  |
|  | *trnV-UAC* | LSC | 39 | 584 | 37 |  |  |
|  | *clpP* | LSC | 71 | 788 | 291 | 658 | 250 |
|  | *petB* | LSC | 6 | 785 | 642 |  |  |
|  | *petD* | LSC | 8 | 737 | 514 |  |  |
|  | *rpl16* | LSC | 9 | 963 | 402 |  |  |
|  | *rpl2* | IR | 394 | 666 | 428 |  |  |
|  | *ndhB* | IR | 775 | 699 | 758 |  |  |
|  | *trnI-GAU* | IR | 42 | 935 | 35 |  |  |
|  | *trnI-GAU* | IR | 42 | 935 | 35 |  |  |
|  | *trnA-UGC* | IR | 38 | 815 | 35 |  |  |
|  | *trnA-UGC* | IR | 38 | 815 | 35 |  |  |
|  | *ndhA* | SSC | 559 | 1125 | 539 |  |  |
|  | *trnA-UGC* | IR | 38 | 815 | 35 |  |  |
|  | *trnA-UGC* | IR | 38 | 815 | 35 |  |  |
|  | *trnI-GAU* | IR | 42 | 935 | 35 |  |  |
|  | *trnI-GAU* | IR | 42 | 935 | 35 |  |  |
|  | *ndhB* | IR | 775 | 699 | 758 |  |  |
|  | *rpl2* | IR | 394 | 666 | 428 |  |  |
| *L. aurea* | *trnK-UUU* | LSC | 46 | 2580 | 35 |  |  |
|  | *rps16* | LSC | 38 | 896 | 208 |  |  |
|  | *trnG-UCC* | LSC | 23 | 690 | 48 |  |  |
|  | *atpF* | LSC | 154 | 1087 | 257 |  |  |
|  | *rpoC1* | LSC | 432 | 751 | 1626 |  |  |
|  | *ycf3* | LSC | 126 | 725 | 231 | 755 | 153 |
|  | *trnL-UAA* | LSC | 30 | 518 | 50 |  |  |
|  | *trnV-UAC* | LSC | 39 | 584 | 37 |  |  |
|  | *clpP* | LSC | 71 | 784 | 291 | 663 | 250 |
|  | *petB* | LSC | 6 | 785 | 642 |  |  |
|  | *petD* | LSC | 8 | 737 | 514 |  |  |
|  | *rpl16* | LSC | 9 | 962 | 402 |  |  |
|  | *rpl2* | IR | 394 | 666 | 428 |  |  |
|  | *ndhB* | IR | 775 | 699 | 758 |  |  |
|  | *trnI-GAU* | IR | 42 | 935 | 35 |  |  |
|  | *trnI-GAU* | IR | 42 | 935 | 35 |  |  |
|  | *trnA-UGC* | IR | 38 | 815 | 35 |  |  |
|  | *trnA-UGC* | IR | 38 | 815 | 35 |  |  |
|  | *ndhA* | SSC | 559 | 1125 | 539 |  |  |
|  | *trnA-UGC* | IR | 38 | 815 | 35 |  |  |
|  | *trnA-UGC* | IR | 38 | 815 | 35 |  |  |
|  | *trnI-GAU* | IR | 42 | 935 | 35 |  |  |
|  | *trnI-GAU* | IR | 42 | 935 | 35 |  |  |
|  | *ndhB* | IR | 775 | 699 | 758 |  |  |
|  | *rpl2* | IR | 394 | 666 | 428 |  |  |
| *L. radiata* | *trnK-UUU* | LSC | 46 | 2581 | 35 |  |  |
|  | *rps16* | LSC | 38 | 891 | 208 |  |  |
|  | *trnG-UCC* | LSC | 23 | 696 | 48 |  |  |
|  | *atpF* | LSC | 154 | 1086 | 257 |  |  |
|  | *rpoC1* | LSC | 432 | 750 | 1626 |  |  |
|  | *ycf3* | LSC | 126 | 725 | 231 | 755 | 153 |
|  | *trnL-UAA* | LSC | 30 | 518 | 50 |  |  |
|  | *trnV-UAC* | LSC | 39 | 584 | 37 |  |  |
|  | *clpP* | LSC | 71 | 783 | 291 | 661 | 250 |
|  | *petB* | LSC | 6 | 783 | 642 |  |  |
|  | *petD* | LSC | 8 | 737 | 514 |  |  |
|  | *rpl16* | LSC | 9 | 962 | 402 |  |  |
|  | *rpl2* | IR | 394 | 666 | 428 |  |  |
|  | *ndhB* | IR | 775 | 699 | 758 |  |  |
|  | *trnI-GAU* | IR | 42 | 935 | 35 |  |  |
|  | *trnI-GAU* | IR | 42 | 935 | 35 |  |  |
|  | *trnA-UGC* | IR | 38 | 815 | 35 |  |  |
|  | *trnA-UGC* | IR | 38 | 815 | 35 |  |  |
|  | *ndhF* | IR/SSC | 2184 | 89 | 57 |  |  |
|  | *ndhA* | SSC | 559 | 1125 | 539 |  |  |
|  | *trnA-UGC* | IR | 38 | 815 | 35 |  |  |
|  | *trnA-UGC* | IR | 38 | 815 | 35 |  |  |
|  | *trnI-GAU* | IR | 42 | 935 | 35 |  |  |
|  | *trnI-GAU* | IR | 42 | 935 | 35 |  |  |
|  | *ndhB* | IR | 775 | 699 | 758 |  |  |
|  | *rpl2* | IR | 394 | 666 | 428 |  |  |
| *L. longituba* | *trnK-UUU* | LSC | 46 | 2577 | 35 |  |  |
|  | *rps16* | LSC | 38 | 887 | 208 |  |  |
|  | *trnG-UCC* | LSC | 23 | 691 | 48 |  |  |
|  | *atpF* | LSC | 154 | 1069 | 257 |  |  |
|  | *rpoC1* | LSC | 432 | 750 | 1626 |  |  |
|  | *ycf3* | LSC | 126 | 725 | 231 | 755 | 153 |
|  | *trnL-UAA* | LSC | 30 | 517 | 50 |  |  |
|  | *trnV-UAC* | LSC | 39 | 584 | 37 |  |  |
|  | *clpP* | LSC | 71 | 788 | 291 | 658 | 250 |
|  | *petB* | LSC | 6 | 785 | 642 |  |  |
|  | *petD* | LSC | 8 | 737 | 514 |  |  |
|  | *rpl16* | LSC | 9 | 963 | 402 |  |  |
|  | *rpl2* | IR | 391 | 666 | 428 |  |  |
|  | *ndhB* | IR | 775 | 699 | 758 |  |  |
|  | *trnI-GAU* | IR | 42 | 935 | 35 |  |  |
|  | *trnI-GAU* | IR | 42 | 935 | 35 |  |  |
|  | *trnA-UGC* | IR | 38 | 815 | 35 |  |  |
|  | *trnA-UGC* | IR | 38 | 815 | 35 |  |  |
|  | *ndhA* | SSC | 559 | 1125 | 539 |  |  |
|  | *trnA-UGC* | IR | 38 | 815 | 35 |  |  |
|  | *trnA-UGC* | IR | 38 | 815 | 35 |  |  |
|  | *trnI-GAU* | IR | 42 | 935 | 35 |  |  |
|  | *trnI-GAU* | IR | 42 | 935 | 35 |  |  |
|  | *ndhB* | IR | 775 | 699 | 758 |  |  |
|  | *rpl2* | IR | 391 | 666 | 428 |  |  |
| *L. sprengeri* | *trnK-UUU* | LSC | 46 | 2581 | 35 |  |  |
|  | *rps16* | LSC | 38 | 887 | 197 |  |  |
|  | *trnG-UCC* | LSC | 23 | 692 | 48 |  |  |
|  | *atpF* | LSC | 154 | 1096 | 257 |  |  |
|  | *rpoC1* | LSC | 432 | 749 | 1626 |  |  |
|  | *ycf3* | LSC | 126 | 725 | 231 | 755 | 153 |
|  | *trnL-UAA* | LSC | 30 | 517 | 50 |  |  |
|  | *trnV-UAC* | LSC | 39 | 584 | 37 |  |  |
|  | *clpP* | LSC | 71 | 788 | 291 | 658 | 250 |
|  | *petB* | LSC | 6 | 785 | 642 |  |  |
|  | *petD* | LSC | 8 | 737 | 514 |  |  |
|  | *rpl16* | LSC | 9 | 963 | 402 |  |  |
|  | *rpl2* | IR | 394 | 666 | 428 |  |  |
|  | *ndhB* | IR | 775 | 699 | 758 |  |  |
|  | *trnI-GAU* | IR | 42 | 935 | 35 |  |  |
|  | *trnI-GAU* | IR | 42 | 935 | 35 |  |  |
|  | *trnA-UGC* | IR | 38 | 815 | 35 |  |  |
|  | *trnA-UGC* | IR | 38 | 815 | 35 |  |  |
|  | *ndhF* | IR/SSC | 2184 | 82 | 6 |  |  |
|  | *ndhA* | SSC | 559 | 1126 | 539 |  |  |
|  | *trnA-UGC* | IR | 38 | 815 | 35 |  |  |
|  | *trnA-UGC* | IR | 38 | 815 | 35 |  |  |
|  | *trnI-GAU* | IR | 42 | 935 | 35 |  |  |
|  | *trnI-GAU* | IR | 42 | 935 | 35 |  |  |
|  | *ndhB* | IR | 775 | 699 | 758 |  |  |
|  | *rpl2* | IR | 394 | 666 | 428 |  |  |
| *L. squamigera* | *trnK-UUU* | LSC | 38 | 2586 | 36 |  |  |
|  | *trnS-CGA* | LSC | 32 | 672 | 61 |  |  |
|  | *atpF* | LSC | 145 | 925 | 410 |  |  |
|  | *rpoC2* | LSC | 4014 | 30 | 108 |  |  |
|  | *rpoC1* | LSC | 432 | 759 | 1617 |  |  |
|  | *ycf3* | LSC | 126 | 728 | 228 | 755 | 153 |
|  | *trnL-UAA* | LSC | 35 | 512 | 50 |  |  |
|  | *trnC-ACA* | LSC | 39 | 565 | 56 |  |  |
|  | *clpP* | LSC | 71 | 785 | 294 | 657 | 250 |
|  | *rpl2* | IR | 388 | 669 | 431 |  |  |
|  | *ndhB* | IR | 775 | 699 | 758 |  |  |
|  | *trnE-UUC* | IR | 32 | 940 | 40 |  |  |
|  | *trnA-UGC* | IR | 37 | 815 | 36 |  |  |
|  | *ndhA* | SSC | 559 | 1125 | 539 |  |  |
|  | *trnA-UGC* | IR | 37 | 815 | 36 |  |  |
|  | *trnE-UUC* | IR | 32 | 940 | 40 |  |  |
|  | *ndhB* | IR | 775 | 699 | 758 |  |  |
|  | *rpl2* | IR | 388 | 669 | 431 |  |  |
