# Supplementary material for: Complete Chloroplast Genomes and Comparative Analyses of L. chinensis, L. anhuiensis, and L. aurea (Amaryllidaceae)
Source: Int J Mol Sci. 2020 Aug 10;21(16):5729. doi: 10.3390/ijms21165729 (PMC7461117; doi:10.3390/ijms21165729)
Supplement: Supplementary file 1 [file ijms-21-05729-s001.zip › ijms-876965-supplementary/Table S2 Chloroplast simple sequence repeats (cpSSRs) of seven Lycoris species.docx]

**Table S2.** Chloroplast simple sequence repeats (cpSSRs) of seven *Lycoris* species

| **Species** | **SSR nr.** | **SSR type** | **SSR** | **Size (bp)** | **Start (bp)** | **End (bp)** | **Region** |
| --- | --- | --- | --- | --- | --- | --- | --- |
| *L. chinensis* | 1 | p1 | (A)10 | 10 | 3596 | 3605 | LSC |
|  | 2 | p1 | (T)10 | 10 | 4644 | 4653 | LSC |
|  | 3 | p1 | (T)10 | 10 | 5293 | 5302 | LSC |
|  | 4 | p1 | (T)11 | 11 | 10615 | 10625 | LSC |
|  | 5 | p1 | (T)14 | 14 | 16987 | 17000 | LSC |
|  | 6 | c | (T)10...(T)10...(T)11 | 139 | 18944 | 19082 | LSC |
|  | 7 | p1 | (T)13 | 13 | 23103 | 23115 | LSC |
|  | 8 | p1 | (T)12 | 12 | 27873 | 27884 | LSC |
|  | 9 | p1 | (A)10 | 10 | 28590 | 28599 | LSC |
|  | 10 | p1 | (A)10 | 10 | 29334 | 29343 | LSC |
|  | 11 | p1 | (A)10 | 10 | 30531 | 30540 | LSC |
|  | 12 | p1 | (T)10 | 10 | 30964 | 30973 | LSC |
|  | 13 | p1 | (T)10 | 10 | 31605 | 31614 | LSC |
|  | 14 | p1 | (A)11 | 11 | 33175 | 33185 | LSC |
|  | 15 | p1 | (A)10 | 10 | 33464 | 33473 | LSC |
|  | 16 | p1 | (T)10 | 10 | 36904 | 36913 | LSC |
|  | 17 | p1 | (A)11 | 11 | 37724 | 37734 | LSC |
|  | 18 | p1 | (A)10 | 10 | 46882 | 46891 | LSC |
|  | 19 | p1 | (T)16 | 16 | 48363 | 48378 | LSC |
|  | 20 | p1 | (A)10 | 10 | 50285 | 50294 | LSC |
|  | 21 | p2 | (TA)7 | 14 | 57377 | 57390 | LSC |
|  | 22 | p1 | (A)10 | 10 | 61528 | 61537 | LSC |
|  | 23 | p1 | (A)10 | 10 | 63745 | 63754 | LSC |
|  | 24 | p1 | (T)10 | 10 | 67997 | 68006 | LSC |
|  | 25 | p1 | (T)11 | 11 | 69061 | 69071 | LSC |
|  | 26 | p1 | (C)10 | 10 | 69823 | 69832 | LSC |
|  | 27 | p1 | (T)15 | 15 | 70167 | 70181 | LSC |
|  | 28 | p1 | (T)10 | 10 | 71056 | 71065 | LSC |
|  | 29 | c | (A)10...(A)10 | 113 | 72863 | 72975 | LSC |
|  | 30 | p1 | (A)11 | 11 | 73927 | 73937 | LSC |
|  | 31 | p1 | (T)10 | 10 | 76574 | 76583 | LSC |
|  | 32 | p1 | (A)10 | 10 | 83739 | 83748 | LSC |
|  | 33 | p1 | (A)10 | 10 | 103155 | 103164 | Ira |
|  | 34 | p1 | (T)10 | 10 | 115431 | 115440 | SSC |
|  | 35 | p1 | (A)13 | 13 | 115841 | 115853 | SSC |
|  | 36 | p1 | (T)10 | 10 | 116338 | 116347 | SSC |
|  | 37 | p1 | (A)12 | 12 | 120851 | 120862 | SSC |
|  | 38 | c | (T)11...(T)11 | 99 | 126942 | 127040 | SSC |
|  | 39 | c | (T)10...(T)11 | 74 | 128093 | 128166 | SSC |
|  | 40 | p1 | (A)12 | 12 | 130547 | 130558 | SSC |
|  | 41 | p1 | (T)10 | 10 | 141780 | 141789 | Irb |
| *L. anhuiensis* | 1 | p1 | (A)10 | 10 | 3596 | 3605 | LSC |
|  | 2 | p1 | (T)10 | 10 | 4644 | 4653 | LSC |
|  | 3 | p1 | (T)11 | 11 | 5293 | 5303 | LSC |
|  | 4 | p1 | (T)11 | 11 | 10616 | 10626 | LSC |
|  | 5 | p1 | (T)15 | 15 | 16988 | 17002 | LSC |
|  | 6 | c | (T)10...(T)10...(T)11 | 139 | 18946 | 19084 | LSC |
|  | 7 | p1 | (T)13 | 13 | 23105 | 23117 | LSC |
|  | 8 | p1 | (T)12 | 12 | 27875 | 27886 | LSC |
|  | 9 | p1 | (A)10 | 10 | 28592 | 28601 | LSC |
|  | 10 | p1 | (A)10 | 10 | 30532 | 30541 | LSC |
|  | 11 | p1 | (T)10 | 10 | 30965 | 30974 | LSC |
|  | 12 | p1 | (T)10 | 10 | 31606 | 31615 | LSC |
|  | 13 | p1 | (A)11 | 11 | 33176 | 33186 | LSC |
|  | 14 | p1 | (A)10 | 10 | 33465 | 33474 | LSC |
|  | 15 | p1 | (T)10 | 10 | 36905 | 36914 | LSC |
|  | 16 | p1 | (A)11 | 11 | 37725 | 37735 | LSC |
|  | 17 | p1 | (A)10 | 10 | 46883 | 46892 | LSC |
|  | 18 | p1 | (T)16 | 16 | 48364 | 48379 | LSC |
|  | 19 | p1 | (A)10 | 10 | 50286 | 50295 | LSC |
|  | 20 | p2 | (TA)7 | 14 | 57378 | 57391 | LSC |
|  | 21 | p1 | (A)10 | 10 | 61529 | 61538 | LSC |
|  | 22 | p1 | (A)10 | 10 | 63746 | 63755 | LSC |
|  | 23 | p1 | (T)10 | 10 | 67998 | 68007 | LSC |
|  | 24 | p1 | (T)11 | 11 | 69067 | 69077 | LSC |
|  | 25 | p1 | (C)10 | 10 | 69829 | 69838 | LSC |
|  | 26 | p1 | (T)15 | 15 | 70173 | 70187 | LSC |
|  | 27 | p1 | (T)10 | 10 | 71062 | 71071 | LSC |
|  | 28 | c | (A)10...(A)10 | 113 | 72869 | 72981 | LSC |
|  | 29 | p1 | (A)11 | 11 | 73933 | 73943 | LSC |
|  | 30 | p1 | (T)10 | 10 | 76580 | 76589 | LSC |
|  | 31 | p1 | (A)10 | 10 | 83745 | 83754 | LSC |
|  | 32 | p1 | (A)10 | 10 | 103161 | 103170 | IRA |
|  | 33 | p1 | (T)10 | 10 | 115437 | 115446 | SSC |
|  | 34 | p1 | (A)13 | 13 | 115847 | 115859 | SSC |
|  | 35 | p1 | (T)10 | 10 | 116344 | 116353 | SSC |
|  | 36 | p1 | (A)12 | 12 | 120857 | 120868 | SSC |
|  | 37 | c | (T)11...(T)11 | 99 | 126948 | 127046 | SSC |
|  | 38 | c | (T)10...(T)11 | 74 | 128099 | 128172 | SSC |
|  | 39 | p1 | (A)12 | 12 | 130553 | 130564 | SSC |
|  | 40 | p1 | (T)10 | 10 | 141786 | 141795 | IRB |
| *L. aurea* | 1 | p1 | (A)12 | 12 | 3583 | 3594 | LSC |
|  | 2 | p1 | (T)10 | 10 | 3705 | 3714 | LSC |
|  | 3 | p1 | (T)10 | 10 | 4634 | 4643 | LSC |
|  | 4 | p1 | (C)12 | 12 | 4881 | 4892 | LSC |
|  | 5 | p1 | (A)10 | 10 | 7481 | 7490 | LSC |
|  | 6 | p1 | (T)12 | 12 | 10623 | 10634 | LSC |
|  | 7 | p1 | (T)10 | 10 | 13051 | 13060 | LSC |
|  | 8 | p1 | (A)11 | 11 | 14154 | 14164 | LSC |
|  | 9 | p1 | (T)12 | 12 | 17023 | 17034 | LSC |
|  | 10 | c | (T)10...(T)10...(T)11 | 139 | 18978 | 19116 | LSC |
|  | 11 | p1 | (T)14 | 14 | 23137 | 23150 | LSC |
|  | 12 | p1 | (A)11 | 11 | 28617 | 28627 | LSC |
|  | 13 | p1 | (A)10 | 10 | 29992 | 30001 | LSC |
|  | 14 | p1 | (A)10 | 10 | 30557 | 30566 | LSC |
|  | 15 | p1 | (T)11 | 11 | 30990 | 31000 | LSC |
|  | 16 | p1 | (T)11 | 11 | 31618 | 31628 | LSC |
|  | 17 | p1 | (A)12 | 12 | 33189 | 33200 | LSC |
|  | 18 | p1 | (A)11 | 11 | 33479 | 33489 | LSC |
|  | 19 | p1 | (A)11 | 11 | 33786 | 33796 | LSC |
|  | 20 | p1 | (T)11 | 11 | 34200 | 34210 | LSC |
|  | 21 | p2 | (TA)7 | 14 | 37257 | 37270 | LSC |
|  | 22 | p1 | (A)10 | 10 | 37754 | 37763 | LSC |
|  | 23 | p1 | (A)11 | 11 | 46928 | 46938 | LSC |
|  | 24 | c | (T)13...(T)12 | 55 | 48411 | 48465 | LSC |
|  | 25 | p1 | (T)10 | 10 | 52923 | 52932 | LSC |
|  | 26 | p1 | (T)10 | 10 | 59818 | 59827 | LSC |
|  | 27 | p1 | (A)10 | 10 | 63807 | 63816 | LSC |
|  | 28 | c | (A)12...(T)10...(A)12 | 124 | 68347 | 68470 | LSC |
|  | 29 | p1 | (T)12 | 12 | 69139 | 69150 | LSC |
|  | 30 | p1 | (T)13 | 13 | 70241 | 70253 | LSC |
|  | 31 | p1 | (T)10 | 10 | 71124 | 71133 | LSC |
|  | 32 | c | (A)13...(A)10 | 27 | 72917 | 72943 | LSC |
|  | 33 | p1 | (T)11 | 11 | 73215 | 73225 | LSC |
|  | 34 | p1 | (A)12 | 12 | 73994 | 74005 | LSC |
|  | 35 | p1 | (T)10 | 10 | 76642 | 76651 | LSC |
|  | 36 | p1 | (G)11 | 11 | 78300 | 78310 | LSC |
|  | 37 | p1 | (A)10 | 10 | 80574 | 80583 | LSC |
|  | 38 | p1 | (A)10 | 10 | 103217 | 103226 | IRA |
|  | 39 | p1 | (T)14 | 14 | 115511 | 115524 | SSC |
|  | 40 | p1 | (A)14 | 14 | 115787 | 115800 | SSC |
|  | 41 | p3 | (ATT)5 | 15 | 116613 | 116627 | SSC |
|  | 42 | p1 | (A)17 | 17 | 120806 | 120822 | SSC |
|  | 43 | c | (T)11...(T)10 | 98 | 126917 | 127014 | SSC |
|  | 44 | c | (T)11...(T)11 | 74 | 128067 | 128140 | SSC |
|  | 45 | p1 | (A)10 | 10 | 130522 | 130531 | SSC |
|  | 46 | p1 | (T)10 | 10 | 141754 | 141763 | IRB |
| *L. radiata* | 1 | p1 | (A)13 | 13 | 3583 | 3595 | LSC |
|  | 2 | p1 | (T)10 | 10 | 3706 | 3715 | LSC |
|  | 3 | p1 | (T)10 | 10 | 4635 | 4644 | LSC |
|  | 4 | p1 | (A)10 | 10 | 7477 | 7486 | LSC |
|  | 5 | p1 | (T)11 | 11 | 8159 | 8169 | LSC |
|  | 6 | p1 | (T)12 | 12 | 10623 | 10634 | LSC |
|  | 7 | p1 | (A)11 | 11 | 14153 | 14163 | LSC |
|  | 8 | p1 | (T)20 | 20 | 17022 | 17041 | LSC |
|  | 9 | c | (T)10...(T)10...(T)11 | 139 | 18985 | 19123 | LSC |
|  | 10 | p1 | (T)13 | 13 | 23144 | 23156 | LSC |
|  | 11 | p1 | (A)10 | 10 | 28624 | 28633 | LSC |
|  | 12 | p1 | (A)10 | 10 | 29367 | 29376 | LSC |
|  | 13 | p1 | (A)10 | 10 | 30000 | 30009 | LSC |
|  | 14 | p1 | (A)11 | 11 | 30565 | 30575 | LSC |
|  | 15 | p1 | (T)10 | 10 | 30999 | 31008 | LSC |
|  | 16 | p1 | (A)13 | 13 | 33195 | 33207 | LSC |
|  | 17 | p1 | (A)11 | 11 | 33486 | 33496 | LSC |
|  | 18 | p1 | (A)10 | 10 | 33793 | 33802 | LSC |
|  | 19 | p1 | (T)10 | 10 | 34206 | 34215 | LSC |
|  | 20 | p1 | (A)10 | 10 | 37753 | 37762 | LSC |
|  | 21 | p1 | (A)12 | 12 | 46927 | 46938 | LSC |
|  | 22 | c | (T)10...(T)12 | 52 | 48411 | 48462 | LSC |
|  | 23 | p1 | (T)10 | 10 | 49699 | 49708 | LSC |
|  | 24 | p1 | (T)11 | 11 | 52921 | 52931 | LSC |
|  | 25 | p2 | (TA)7 | 14 | 57436 | 57449 | LSC |
|  | 26 | p1 | (T)10 | 10 | 59821 | 59830 | LSC |
|  | 27 | p1 | (A)10 | 10 | 63811 | 63820 | LSC |
|  | 28 | c | (A)13...(T)10...(A)11 | 124 | 68351 | 68474 | LSC |
|  | 29 | p1 | (T)10 | 10 | 69143 | 69152 | LSC |
|  | 30 | p1 | (C)12 | 12 | 69900 | 69911 | LSC |
|  | 31 | p1 | (T)13 | 13 | 70246 | 70258 | LSC |
|  | 32 | p1 | (T)10 | 10 | 71129 | 71138 | LSC |
|  | 33 | c | (A)11...(A)10 | 25 | 72922 | 72946 | LSC |
|  | 34 | p1 | (T)11 | 11 | 73218 | 73228 | LSC |
|  | 35 | p1 | (A)12 | 12 | 73996 | 74007 | LSC |
|  | 36 | p1 | (T)13 | 13 | 76644 | 76656 | LSC |
|  | 37 | p1 | (A)10 | 10 | 103218 | 103227 | IRA |
|  | 38 | p1 | (T)14 | 14 | 115583 | 115596 | SSC |
|  | 39 | p1 | (A)13 | 13 | 115863 | 115875 | SSC |
|  | 40 | p1 | (A)10 | 10 | 116007 | 116016 | SSC |
|  | 41 | p3 | (ATT)5 | 15 | 116689 | 116703 | SSC |
|  | 42 | p1 | (A)16 | 16 | 120882 | 120897 | SSC |
|  | 43 | p1 | (T)11 | 11 | 126972 | 126982 | SSC |
|  | 44 | c | (T)11...(T)11 | 74 | 128035 | 128108 | SSC |
|  | 45 | p1 | (A)10 | 10 | 130490 | 130499 | SSC |
|  | 46 | p1 | (T)10 | 10 | 141722 | 141731 | IRB |
| *L. longituba* | 1 | p1 | (A)10 | 10 | 3596 | 3605 | LSC |
|  | 2 | p1 | (T)10 | 10 | 4644 | 4653 | LSC |
|  | 3 | p1 | (T)10 | 10 | 5293 | 5302 | LSC |
|  | 4 | p1 | (T)11 | 11 | 10615 | 10625 | LSC |
|  | 5 | p1 | (T)14 | 14 | 16987 | 17000 | LSC |
|  | 6 | c | (T)10...(T)10...(T)11 | 139 | 18944 | 19082 | LSC |
|  | 7 | p1 | (T)13 | 13 | 23103 | 23115 | LSC |
|  | 8 | p1 | (T)12 | 12 | 27873 | 27884 | LSC |
|  | 9 | p1 | (A)10 | 10 | 28590 | 28599 | LSC |
|  | 10 | p1 | (A)10 | 10 | 29334 | 29343 | LSC |
|  | 11 | p1 | (A)10 | 10 | 30531 | 30540 | LSC |
|  | 12 | p1 | (T)10 | 10 | 30964 | 30973 | LSC |
|  | 13 | p1 | (T)10 | 10 | 31605 | 31614 | LSC |
|  | 14 | p1 | (A)11 | 11 | 33175 | 33185 | LSC |
|  | 15 | p1 | (A)10 | 10 | 33464 | 33473 | LSC |
|  | 16 | p1 | (T)10 | 10 | 36904 | 36913 | LSC |
|  | 17 | p1 | (A)11 | 11 | 37724 | 37734 | LSC |
|  | 18 | p1 | (A)10 | 10 | 46882 | 46891 | LSC |
|  | 19 | p1 | (T)16 | 16 | 48363 | 48378 | LSC |
|  | 20 | p1 | (A)10 | 10 | 50285 | 50294 | LSC |
|  | 21 | p2 | (TA)7 | 14 | 57377 | 57390 | LSC |
|  | 22 | p1 | (A)10 | 10 | 61528 | 61537 | LSC |
|  | 23 | p1 | (A)10 | 10 | 63745 | 63754 | LSC |
|  | 24 | p1 | (T)10 | 10 | 67997 | 68006 | LSC |
|  | 25 | p1 | (T)11 | 11 | 69061 | 69071 | LSC |
|  | 26 | p1 | (C)10 | 10 | 69823 | 69832 | LSC |
|  | 27 | p1 | (T)15 | 15 | 70167 | 70181 | LSC |
|  | 28 | p1 | (T)10 | 10 | 71056 | 71065 | LSC |
|  | 29 | c | (A)10...(A)10 | 113 | 72863 | 72975 | LSC |
|  | 30 | p1 | (A)11 | 11 | 73927 | 73937 | LSC |
|  | 31 | p1 | (T)10 | 10 | 76574 | 76583 | LSC |
|  | 32 | p1 | (A)10 | 10 | 83739 | 83748 | LSC |
|  | 33 | p1 | (A)10 | 10 | 103155 | 103164 | IRA |
|  | 34 | p1 | (T)10 | 10 | 115431 | 115440 | SSC |
|  | 35 | p1 | (A)13 | 13 | 115841 | 115853 | SSC |
|  | 36 | p1 | (T)10 | 10 | 116338 | 116347 | SSC |
|  | 37 | p1 | (A)12 | 12 | 120851 | 120862 | SSC |
|  | 38 | c | (T)11...(T)11 | 99 | 126942 | 127040 | SSC |
|  | 39 | c | (T)10...(T)11 | 74 | 128093 | 128166 | SSC |
|  | 40 | p1 | (A)12 | 12 | 130547 | 130558 | SSC |
|  | 41 | p1 | (T)10 | 10 | 141780 | 141789 | IRB |
| *L. sprengeri* | 1 | p1 | (A)13 | 13 | 3583 | 3595 | LSC |
|  | 2 | p1 | (T)10 | 10 | 3706 | 3715 | LSC |
|  | 3 | p1 | (T)10 | 10 | 10388 | 10397 | LSC |
|  | 4 | p1 | (T)10 | 10 | 10598 | 10607 | LSC |
|  | 5 | p1 | (T)12 | 12 | 13373 | 13384 | LSC |
|  | 6 | p1 | (A)11 | 11 | 14130 | 14140 | LSC |
|  | 7 | p1 | (T)12 | 12 | 16998 | 17009 | LSC |
|  | 8 | c | (T)10...(T)10...(T)11 | 139 | 18953 | 19091 | LSC |
|  | 9 | p1 | (T)12 | 12 | 23112 | 23123 | LSC |
|  | 10 | p1 | (A)10 | 10 | 28593 | 28602 | LSC |
|  | 11 | p1 | (A)10 | 10 | 29313 | 29322 | LSC |
|  | 12 | p1 | (A)11 | 11 | 30509 | 30519 | LSC |
|  | 13 | p1 | (T)10 | 10 | 30943 | 30952 | LSC |
|  | 14 | p1 | (T)10 | 10 | 31588 | 31597 | LSC |
|  | 15 | p1 | (A)12 | 12 | 33158 | 33169 | LSC |
|  | 16 | p1 | (T)10 | 10 | 36886 | 36895 | LSC |
|  | 17 | p1 | (A)10 | 10 | 46853 | 46862 | LSC |
|  | 18 | c | (T)14...(T)10 | 54 | 48335 | 48388 | LSC |
|  | 19 | p1 | (T)11 | 11 | 52840 | 52850 | LSC |
|  | 20 | p2 | (TA)9 | 18 | 57360 | 57377 | LSC |
|  | 21 | p1 | (A)10 | 10 | 61515 | 61524 | LSC |
|  | 22 | p1 | (A)15 | 15 | 63758 | 63772 | LSC |
|  | 23 | p1 | (A)12 | 12 | 66211 | 66222 | LSC |
|  | 24 | p1 | (T)10 | 10 | 68020 | 68029 | LSC |
|  | 25 | p1 | (A)10 | 10 | 68305 | 68314 | LSC |
|  | 26 | p1 | (T)11 | 11 | 69090 | 69100 | LSC |
|  | 27 | p1 | (T)12 | 12 | 70194 | 70205 | LSC |
|  | 28 | p1 | (T)12 | 12 | 71080 | 71091 | LSC |
|  | 29 | p1 | (T)11 | 11 | 73169 | 73179 | LSC |
|  | 30 | p1 | (A)11 | 11 | 73953 | 73963 | LSC |
|  | 31 | p1 | (T)10 | 10 | 74693 | 74702 | LSC |
|  | 32 | p1 | (T)11 | 11 | 76604 | 76614 | LSC |
|  | 33 | p1 | (A)10 | 10 | 83770 | 83779 | LSC |
|  | 34 | p1 | (A)10 | 10 | 103209 | 103218 | IRA |
|  | 35 | p1 | (A)12 | 12 | 116001 | 116012 | SSC |
|  | 36 | p3 | (ATT)5 | 15 | 116824 | 116838 | SSC |
|  | 37 | p1 | (A)13 | 13 | 121010 | 121022 | SSC |
|  | 38 | c | (T)10...(T)13 | 112 | 127109 | 127220 | SSC |
|  | 39 | c | (T)10...(T)11 | 74 | 128273 | 128346 | SSC |
|  | 40 | p1 | (A)10 | 10 | 130728 | 130737 | SSC |
|  | 41 | p1 | (T)10 | 10 | 141960 | 141969 | IRB |
| *L. squamigera* | 1 | p1 | (A)10 | 10 | 3596 | 3605 | LSC |
|  | 2 | p1 | (T)10 | 10 | 4644 | 4653 | LSC |
|  | 3 | p1 | (A)10 | 10 | 7488 | 7497 | LSC |
|  | 4 | p1 | (T)11 | 11 | 10615 | 10625 | LSC |
|  | 5 | p1 | (T)17 | 17 | 16987 | 17003 | LSC |
|  | 6 | c | (T)10...(T)10...(T)11 | 139 | 18947 | 19085 | LSC |
|  | 7 | p1 | (T)13 | 13 | 23106 | 23118 | LSC |
|  | 8 | p1 | (T)11 | 11 | 27876 | 27886 | LSC |
|  | 9 | p1 | (A)10 | 10 | 28592 | 28601 | LSC |
|  | 10 | p1 | (A)11 | 11 | 29336 | 29346 | LSC |
|  | 11 | p1 | (A)10 | 10 | 30534 | 30543 | LSC |
|  | 12 | p1 | (T)10 | 10 | 30967 | 30976 | LSC |
|  | 13 | p1 | (A)11 | 11 | 33177 | 33187 | LSC |
|  | 14 | p1 | (A)11 | 11 | 33466 | 33476 | LSC |
|  | 15 | p1 | (T)10 | 10 | 36907 | 36916 | LSC |
|  | 16 | p1 | (A)10 | 10 | 37727 | 37736 | LSC |
|  | 17 | p1 | (A)10 | 10 | 46864 | 46873 | LSC |
|  | 18 | p1 | (T)15 | 15 | 48345 | 48359 | LSC |
|  | 19 | p1 | (A)12 | 12 | 50266 | 50277 | LSC |
|  | 20 | p1 | (T)12 | 12 | 52847 | 52858 | LSC |
|  | 21 | p2 | (TA)7 | 14 | 57363 | 57376 | LSC |
|  | 22 | p1 | (A)10 | 10 | 61514 | 61523 | LSC |
|  | 23 | p1 | (A)10 | 10 | 63716 | 63725 | LSC |
|  | 24 | p1 | (T)10 | 10 | 67968 | 67977 | LSC |
|  | 25 | p1 | (T)10 | 10 | 69037 | 69046 | LSC |
|  | 26 | p1 | (T)15 | 15 | 70140 | 70154 | LSC |
|  | 27 | p1 | (T)10 | 10 | 71029 | 71038 | LSC |
|  | 28 | p1 | (T)10 | 10 | 73116 | 73125 | LSC |
|  | 29 | p1 | (A)11 | 11 | 73899 | 73909 | LSC |
|  | 30 | p1 | (A)10 | 10 | 83711 | 83720 | LSC |
|  | 31 | p1 | (A)10 | 10 | 103127 | 103136 | IRA |
|  | 32 | p1 | (T)10 | 10 | 115403 | 115412 | SSC |
|  | 33 | p1 | (A)12 | 12 | 115813 | 115824 | SSC |
|  | 34 | p1 | (T)10 | 10 | 116309 | 116318 | SSC |
|  | 35 | p3 | (ATT)5 | 15 | 116638 | 116652 | SSC |
|  | 36 | p1 | (A)13 | 13 | 120829 | 120841 | SSC |
|  | 37 | c | (T)10...(T)10 | 97 | 126919 | 127015 | SSC |
|  | 38 | c | (T)10...(T)11 | 74 | 128068 | 128141 | SSC |
|  | 39 | p1 | (A)11 | 11 | 130523 | 130533 | SSC |
|  | 40 | p1 | (T)10 | 10 | 141755 | 141764 | IRB |
