# Supplementary material for: Complete Chloroplast Genomes and Comparative Analyses of L. chinensis, L. anhuiensis, and L. aurea (Amaryllidaceae)
Source: Int J Mol Sci. 2020 Aug 10;21(16):5729. doi: 10.3390/ijms21165729 (PMC7461117; doi:10.3390/ijms21165729)
Supplement: Supplementary file 1 [file ijms-21-05729-s001.zip › ijms-876965-supplementary/Table S3 Relative synonymous codon usage (RSCU) in the Lycoris chloroplast genomes.docx]

**Table S3.** Relative synonymous codon usage (RSCU) in the *Lycoris* chloroplast genomes.

| **Amino acid** | **Codon** | **Species** | | | | | | | | | | | | | |
| --- | --- | --- | --- | --- | --- | --- | --- | --- | --- | --- | --- | --- | --- | --- | --- |
|  |  | ***L. chinensis*** | | ***L. anhuiensis*** | | ***L. aurea*** | | ***L. radiata*** | | ***L. longituba*** | | ***L. sprengeri*** | | ***L. squamigera*** | |
|  |  | **Number** | **RSCU** | **Number** | **RSCU** | **Number** | **RSCU** | **Number** | **RSCU** | **Number** | **RSCU** | **Number** | **RSCU** | **Number** | **RSCU** |
| Ala | GCA | 711 | 1.13 | 711 | 1.13 | 715 | 1.13 | 693 | 1.14 | 711 | 1.13 | 687 | 1.13 | 592 | 1.13 |
| Ala | GCC | 370 | 0.59 | 370 | 0.59 | 370 | 0.59 | 358 | 0.59 | 370 | 0.59 | 362 | 0.60 | 315 | 0.60 |
| Ala | GCG | 286 | 0.46 | 286 | 0.46 | 290 | 0.46 | 276 | 0.46 | 286 | 0.46 | 279 | 0.46 | 228 | 0.43 |
| Ala | GCT | 1142 | 1.82 | 1142 | 1.82 | 1148 | 1.82 | 1098 | 1.81 | 1142 | 1.82 | 1100 | 1.81 | 968 | 1.84 |
| Cys | TGC | 175 | 0.56 | 175 | 0.56 | 176 | 0.56 | 172 | 0.56 | 175 | 0.56 | 176 | 0.57 | 110 | 0.54 |
| Cys | TGT | 447 | 1.44 | 447 | 1.44 | 448 | 1.44 | 438 | 1.44 | 447 | 1.44 | 440 | 1.43 | 296 | 1.46 |
| Asp | GAC | 406 | 0.40 | 406 | 0.40 | 410 | 0.40 | 394 | 0.40 | 406 | 0.40 | 391 | 0.39 | 261 | 0.44 |
| Asp | GAT | 1630 | 1.60 | 1630 | 1.60 | 1654 | 1.60 | 1598 | 1.60 | 1630 | 1.60 | 1597 | 1.61 | 932 | 1.56 |
| Glu | GAA | 1928 | 1.46 | 1928 | 1.46 | 1986 | 1.47 | 1914 | 1.46 | 1928 | 1.46 | 1902 | 1.46 | 1266 | 1.51 |
| Glu | GAG | 716 | 0.54 | 716 | 0.54 | 722 | 0.53 | 710 | 0.54 | 716 | 0.54 | 704 | 0.54 | 408 | 0.49 |
| Phe | TTC | 1135 | 0.77 | 1135 | 0.77 | 1130 | 0.78 | 1102 | 0.79 | 1135 | 0.77 | 1093 | 0.78 | 692 | 0.72 |
| Phe | TTT | 1797 | 1.23 | 1797 | 1.23 | 1785 | 1.22 | 1703 | 1.21 | 1797 | 1.23 | 1698 | 1.22 | 1240 | 1.28 |
| Gly | GGA | 1281 | 1.60 | 1281 | 1.60 | 1273 | 1.60 | 1215 | 1.57 | 1281 | 1.60 | 1226 | 1.58 | 960 | 1.53 |
| Gly | GGC | 315 | 0.39 | 315 | 0.39 | 315 | 0.40 | 311 | 0.40 | 315 | 0.39 | 310 | 0.40 | 270 | 0.43 |
| Gly | GGG | 565 | 0.71 | 565 | 0.71 | 561 | 0.71 | 553 | 0.72 | 567 | 0.71 | 557 | 0.72 | 412 | 0.66 |
| Gly | GGT | 1041 | 1.30 | 1041 | 1.30 | 1033 | 1.30 | 1009 | 1.31 | 1041 | 1.30 | 1005 | 1.30 | 860 | 1.37 |
| His | CAC | 329 | 0.52 | 329 | 0.52 | 331 | 0.52 | 319 | 0.51 | 329 | 0.52 | 319 | 0.51 | 214 | 0.50 |
| His | CAT | 946 | 1.48 | 946 | 1.48 | 946 | 1.48 | 928 | 1.49 | 946 | 1.48 | 926 | 1.49 | 638 | 1.50 |
| Ile | ATA | 1303 | 0.93 | 1303 | 0.93 | 1317 | 0.94 | 1245 | 0.93 | 1303 | 0.93 | 1245 | 0.93 | 856 | 0.91 |
| Ile | ATC | 881 | 0.63 | 881 | 0.63 | 876 | 0.63 | 864 | 0.64 | 881 | 0.63 | 860 | 0.64 | 584 | 0.62 |
| Ile | ATT | 2002 | 1.43 | 2002 | 1.43 | 2010 | 1.43 | 1920 | 1.43 | 2002 | 1.43 | 1921 | 1.43 | 1391 | 1.47 |
| Lys | AAA | 1938 | 1.43 | 1938 | 1.43 | 1986 | 1.44 | 1918 | 1.44 | 1938 | 1.71 | 1875 | 1.43 | 1133 | 1.49 |
| Lys | AAG | 765 | 0.57 | 765 | 0.57 | 763 | 0.56 | 749 | 0.56 | 765 | 0.68 | 742 | 0.57 | 388 | 0.51 |
| Leu | CTA | 693 | 0.82 | 693 | 0.82 | 700 | 0.83 | 680 | 0.83 | 693 | 0.82 | 673 | 0.83 | 446 | 0.78 |
| Leu | CTC | 372 | 0.44 | 372 | 0.44 | 364 | 0.43 | 362 | 0.44 | 372 | 0.44 | 356 | 0.44 | 236 | 0.41 |
| Leu | CTG | 339 | 0.40 | 339 | 0.40 | 339 | 0.40 | 331 | 0.41 | 339 | 0.40 | 325 | 0.40 | 216 | 0.38 |
| Leu | CTT | 1021 | 1.21 | 1021 | 1.21 | 1033 | 1.22 | 991 | 1.21 | 1021 | 1.21 | 983 | 1.22 | 683 | 1.19 |
| Leu | TTA | 1483 | 1.76 | 1483 | 1.76 | 1465 | 1.73 | 1407 | 1.72 | 1483 | 1.76 | 1396 | 1.73 | 1128 | 1.97 |
| Leu | TTG | 1159 | 1.37 | 1159 | 1.37 | 1170 | 1.38 | 1126 | 1.38 | 1159 | 1.37 | 1112 | 1.38 | 725 | 1.27 |
| Met | ATG | 1155 | 1.00 | 1155 | 1.00 | 1161 | 1.00 | 1107 | 1.00 | 1155 | 1.00 | 1095 | 1.00 | 830 | 1.00 |
| Asn | AAC | 585 | 0.49 | 585 | 0.49 | 595 | 0.50 | 579 | 0.51 | 585 | 0.49 | 579 | 0.51 | 349 | 0.51 |
| Asn | AAT | 1792 | 1.51 | 1792 | 1.51 | 1774 | 1.50 | 1714 | 1.49 | 1792 | 1.51 | 1704 | 1.49 | 1022 | 1.49 |
| Pro | CCA | 575 | 1.15 | 575 | 1.15 | 577 | 1.14 | 549 | 1.12 | 575 | 1.15 | 547 | 1.12 | 395 | 1.12 |
| Pro | CCC | 466 | 0.93 | 466 | 0.93 | 466 | 0.92 | 458 | 0.93 | 466 | 0.93 | 449 | 0.92 | 327 | 0.93 |
| Pro | CCG | 272 | 0.54 | 272 | 0.54 | 280 | 0.55 | 274 | 0.56 | 272 | 0.54 | 275 | 0.56 | 171 | 0.49 |
| Pro | CCT | 694 | 1.38 | 694 | 1.38 | 704 | 1.39 | 682 | 1.39 | 694 | 1.38 | 680 | 1.39 | 517 | 1.47 |
| Gln | CAA | 1306 | 1.48 | 1306 | 1.48 | 1318 | 1.43 | 1276 | 1.47 | 1306 | 1.48 | 1272 | 1.47 | 837 | 1.47 |
| Gln | CAG | 462 | 0.52 | 462 | 0.52 | 460 | 0.50 | 456 | 0.53 | 462 | 0.52 | 457 | 0.53 | 299 | 0.53 |
| Arg | AGA | 965 | 1.86 | 965 | 1.86 | 971 | 1.87 | 947 | 1.86 | 965 | 1.86 | 943 | 1.86 | 602 | 1.75 |
| Arg | AGG | 367 | 0.71 | 367 | 0.71 | 367 | 0.71 | 365 | 0.72 | 367 | 0.71 | 364 | 0.72 | 224 | 0.65 |
| Arg | CGA | 651 | 1.25 | 651 | 1.25 | 643 | 1.24 | 639 | 1.25 | 651 | 1.25 | 635 | 1.25 | 421 | 1.22 |
| Arg | CGC | 214 | 0.41 | 214 | 0.41 | 220 | 0.42 | 216 | 0.42 | 214 | 0.41 | 214 | 0.42 | 148 | 0.43 |
| Arg | CGG | 269 | 0.52 | 269 | 0.52 | 267 | 0.51 | 259 | 0.51 | 269 | 0.52 | 253 | 0.50 | 151 | 0.44 |
| Arg | CGT | 647 | 1.25 | 647 | 1.25 | 645 | 1.24 | 633 | 1.24 | 647 | 1.25 | 636 | 1.25 | 520 | 1.51 |
| Ser | AGC | 237 | 0.36 | 237 | 0.36 | 234 | 0.37 | 232 | 0.36 | 235 | 0.35 | 231 | 0.36 | 171 | 0.41 |
| Ser | AGT | 775 | 1.16 | 775 | 1.16 | 779 | 1.24 | 747 | 1.16 | 777 | 1.17 | 741 | 1.16 | 548 | 1.32 |
| Ser | TCA | 806 | 1.21 | 806 | 1.21 | 808 | 1.29 | 784 | 1.21 | 806 | 1.21 | 775 | 1.21 | 457 | 1.10 |
| Ser | TCC | 699 | 1.05 | 699 | 1.05 | 702 | 1.12 | 676 | 1.05 | 699 | 1.05 | 669 | 1.04 | 409 | 0.98 |
| Ser | TCG | 397 | 0.60 | 397 | 0.60 | 409 | 0.65 | 387 | 0.60 | 397 | 0.60 | 387 | 0.60 | 228 | 0.55 |
| Ser | TCT | 1087 | 1.63 | 1087 | 1.63 | 1104 | 1.76 | 1046 | 1.62 | 1087 | 1.63 | 1039 | 1.62 | 687 | 1.65 |
| Thr | ACA | 714 | 1.14 | 714 | 1.14 | 730 | 1.17 | 692 | 1.14 | 714 | 1.14 | 705 | 1.16 | 487 | 1.12 |
| Thr | ACC | 478 | 0.77 | 478 | 0.77 | 487 | 0.78 | 473 | 0.78 | 478 | 0.77 | 473 | 0.78 | 333 | 0.77 |
| Thr | ACG | 298 | 0.48 | 298 | 0.48 | 296 | 0.47 | 294 | 0.48 | 298 | 0.48 | 290 | 0.48 | 167 | 0.39 |
| Thr | ACT | 1009 | 1.62 | 1009 | 1.62 | 993 | 1.58 | 967 | 1.59 | 1009 | 1.62 | 957 | 1.58 | 746 | 1.72 |
| Val | GTA | 943 | 1.45 | 943 | 1.45 | 932 | 1.44 | 910 | 1.44 | 943 | 1.45 | 905 | 1.44 | 748 | 1.54 |
| Val | GTC | 370 | 0.57 | 370 | 0.57 | 372 | 0.57 | 370 | 0.58 | 370 | 0.57 | 358 | 0.57 | 245 | 0.50 |
| Val | GTG | 363 | 0.56 | 363 | 0.56 | 357 | 0.55 | 353 | 0.56 | 363 | 0.56 | 351 | 0.56 | 247 | 0.51 |
| Val | GTT | 932 | 1.43 | 932 | 1.43 | 930 | 1.44 | 902 | 1.42 | 932 | 1.43 | 902 | 1.43 | 702 | 1.45 |
| Trp | TGG | 898 | 1.00 | 898 | 1.00 | 906 | 1.00 | 878 | 1.00 | 896 | 1.00 | 863 | 1.00 | 641 | 1.00 |
| Tyr | TAC | 388 | 0.42 | 388 | 0.42 | 384 | 0.42 | 374 | 0.42 | 386 | 0.42 | 374 | 0.42 | 260 | 0.42 |
| Tyr | TAT | 1459 | 1.58 | 1459 | 1.58 | 1449 | 1.58 | 1393 | 1.58 | 1459 | 1.58 | 1400 | 1.58 | 984 | 1.58 |
| Stop | TAA | 128 | 1.10 | 128 | 1.10 | 122 | 1.06 | 124 | 1.08 | 128 | 1.10 | 128 | 1.10 | 114 | 1.07 |
| Stop | TAG | 117 | 1.01 | 117 | 1.01 | 116 | 1.01 | 116 | 1.01 | 117 | 1.01 | 116 | 1.00 | 106 | 1.00 |
| Stop | TGA | 104 | 0.89 | 104 | 0.89 | 108 | 0.94 | 106 | 0.92 | 104 | 0.89 | 104 | 0.90 | 99 | 0.93 |
| Total |  | 49798 | 64 | 49798 | 64 | 49982 | 64 | 48362 | 64 | 49796 | 64 | 48131 | 64 | 33640 | 64 |

RSCU: Relative Synonymous Codon Usage.
