# Supplementary material for: Complete Chloroplast Genomes and Comparative Analyses of L. chinensis, L. anhuiensis, and L. aurea (Amaryllidaceae)
Source: Int J Mol Sci. 2020 Aug 10;21(16):5729. doi: 10.3390/ijms21165729 (PMC7461117; doi:10.3390/ijms21165729)
Supplement: Supplementary file 1 [file ijms-21-05729-s001.zip › ijms-876965-supplementary/Table S4 The GenBank accession numbers of complete chloroplast genome sequences used for phylogenetic analysis.docx]

**Table S4.** The GenBank accession numbers of complete chloroplast genome sequences used for phylogenetic analysis.

| **GenBank accession** | **Species** |
| --- | --- |
| MN158120 | *Lycoris radiata* |
| MN158985 | *Lycoris aurea* |
| MN096601 | *Lycoris longituba* |
| MH118290 | *Lycoris squamigera* |
| MT700549 | *Lycoris chinensis* |
| MT700550 | *Lycoris anhuiensis* |
| MN158986 | *Lycoris sprengeri* |
| MH706763 | *Narcissus poeticus* |
| NC_024813 | *Allium cepa* |
| MG739457 | *Allium prattii* |
| NC_035971 | *Agapanthus coddii* |
| KX931460 | *Hosta ventricosa* |
| KX931469 | *Yucca schidigera* |
| NC_035588 | *Lilium brownii* |
| KR260986 | *Elleanthus sodiroi* |
| KM014691 | *Iris gatesii* |
